# Radiation therapy causes a STING and MyD88-independent upregulation of CD80 and CD86 in macrophages and monocytes that limits tumor control

**DOI:** 10.1007/s00262-025-04272-0

**Published:** 2025-12-19

**Authors:** Aanchal Preet Kaur, Jason R. Baird, Alejandro F. Alice, Gwen Kramer, David Friedman, Eva Moran, Marka Crittenden, Michael J. Gough

**Affiliations:** 1 Providence Cancer Institute, Earle A. Chiles Research Institute, 4805 NE Glisan St., Portland, OR 97213 USA; 2 The Oregon Clinic, Portland, OR 97213 USA

**Keywords:** Radiation, Tumor, Macrophage, CD80, Treg

## Abstract

In preclinical models, optimum tumor control by radiation therapy incorporates CD8 T cell control of residual cancer cells. Tumor control by these CD8 T cells is negatively regulated by myeloid and T regulatory cell expansions in the tumor following radiation treatment. We demonstrate using ex vivo 3D tumor models and novel in vivo cell tracking models that radiation results in upregulation of the costimulatory molecules CD80 and CD86 on tumor macrophages and on monocytes that enter the tumor following radiation. This regulation of CD80 is not dependent on adjuvant signaling via MyD88 or STING in the myeloid cells. While we would anticipate that CD80 and CD86 would have a positive impact on anti-tumor immunity, we demonstrate that blockade of CD80 and CD86 signaling limits CD4 infiltrates in tumors and prevents the Treg expansion caused by radiation treatment. Importantly, this treatment results in improved tumor control following radiation therapy. These data link the inflammatory activation of myeloid cells in irradiated tumors to the Treg expansion following radiation and ensuing suppression of anti-tumor immunity.

**Supplementary Information:**

The online version contains supplementary material available at 10.1007/s00262-025-04272-0.

## Introduction

Radiation therapy targeted to the tumor impacts cancer cells and the various tumor infiltrating cells within the field of radiation. Irradiated cancer cells can directly interact with phagocytic cells to alter their phenotype [1, 2] and also release endogenous adjuvants with potential pro- and anti-inflammatory consequences [3, 4]. These agents can directly impact immune cells within the tumor environment but also regulate recruitment of new cells into the tumor. Radiation treatment results in an increase in the proportion of myeloid cells and T regulatory cells that negatively regulate effector CD8 T cell control of residual cancer cells [2, 5–8]. This movement of cells also means that it can be difficult to assign treatment-mediated shifts in cell phenotypes to new infiltration, in situ activation, or some combination of the two.

To isolate the impacts of radiation therapy on pre-existing versus infiltrating myeloid cells, we developed two models. Firstly, to isolate the impact of radiation on pre-existing macrophages we employed an ex vivo 3D model employing cancer spheroids, which was validated with tumors treated in vivo. Secondly, to distinguish the effect of the post-RT environment on infiltrating myeloid cells, we developed an in vivo cell tracking system. Using these models, we demonstrate that radiation results in significant upregulation of the costimulatory molecules CD80 and CD86 on monocytes and macrophages in the tumor, and on monocytes that are recruited to the tumor following radiation. Finally, we demonstrated that blockade of CD80 and CD86 improved the response to radiation, associated with decreased Treg in the tumor. These data demonstrate that myeloid cells in the post-radiation tumor are activated to upregulate costimulatory molecules that drive the Treg expansion observed post-RT. These data provide a mechanistic link explaining the signals driving myeloid activation and the Treg expansion in the tumor environment following radiation, leading to suppression of anti-tumor immunity.

## Materials and methods

### Animals and cell lines

Animal protocols were approved by and performed according to the requirements of the Earle A. Chiles Research Institute (EACRI) Institutional Animal Care and Use Committee (Animal Welfare Assurance No. D16-00526). Experiments were performed according to ARRIVE guidelines. For tissue harvest the mice were euthanized by CO2 inhalation followed by cervical dislocation. Congenic CD45.1 (Jax# 002014), CD45.2 mice (Jax # 000664), IFNAR1^−/−^ (#028288), and Sting1^−/−^ (*Goldenticket* Stock# 017537) C57BL/6 mice were obtained from The Jackson Laboratories. *Lyz2-Cre* (Stock #004781) [9], and *Myd88*^*fl/fl*^ (Stock #008888) [10] mice were obtained from The Jackson Laboratories and bred to generate *Lyz2-Cre Myd88*^*fl/fl*^ mice as previously described [3]. Kaede transgenic mice were kindly provided by Dr. Amanda Lund at Oregon Health & Science University as previously described [11]. Experiments were performed with 8–12-week-old mice with 4–8 female mice per group. The MC38 colorectal carcinoma line was obtained from Dr. Kristina Young at EACRI. The Panc02-SIY pancreatic adenocarcinoma line expressing the model antigen SIY was kindly provided by Dr. Ralph Weichselbaum at the University of Chicago. Pathogen and mycoplasma contamination testing were performed on all cell lines using the IMPACT II Mouse PCR Profiling from IDEXX BioAnalytics.

### Spheroid formation and mixed bone marrow-derived macrophage co-culture assays

Cancer cell suspensions were seeded at 2500 cells/well in 50 μL in a 96-well ultra-low attachment plates (S-Bio, #MS-9096UZ), spun at 300xg for 5 min and incubated overnight to form spheroids. The radiation treatment plates were exposed to 12 Gy of radiation using an X-ray irradiator (XStrahl). For hypoxia experiments, Image-iT reagent (Thermo Fisher Scientific, #I14834) was added 24 h post time of irradiation as per manufacturer’s recommendation. All imaging was performed using the live cell imaging tool, CellCyteX, at 4X magnification.

Bone marrow-derived macrophages (BMM0) were generated from bone marrow isolated from femurs and tibias of mice as previously described [12], with M-CSF added on d0 and d3 and cell harvest on d7. For co-culture assays, tumor cells were stained with Hoechst 33,342 dye as per manufacturer’s recommendation. Stained tumor cells were plated with BMM0 in a 1:1 ratio, and co-cultures were allowed to form spheroids as above.

For analysis, cells were washed and disrupted using 2 mM ice-cold EDTA solution with 30–45 min incubation on ice with continuous pipetting at intervals of 10 min. The single cells were then stained and fixed for flow cytometry.

### In vivo tumor treatments

2 × 10^5^ MC38 or 2 × 10^6^ Panc02-SIY cells were injected subcutaneously in the right flank of mice. On day 14, mice were randomized to receive no treatment or treatment with CT-guided radiation using the Small Animal Radiation Research Platform (SARRP) from XStrahl as previously described [11]. Dosimetry for the CT-guided radiation treatment was performed using MuriSlice software by XStrahl. A single dose of 12 Gy was delivered to an isocenter within the tumor using a 10 mm × 10 mm collimator at a 45° beam angle to minimize irradiation of normal tissues. For dual tumor studies, both tumors were established simultaneously on both flanks and only one tumor received radiation therapy.

For in vivo cell tracking experiments, mice were injected intravenously (IV) with Latex green fluorescent beads (Fluoresbrite YG Carboxylate Microspheres 0.50 µm, Cat# 15,700–10, Polysciences). Mice received 100 µL of a 1:10 PBS dilution of the factory stock solution or vector control retro-orbitally.

As an alternative approach to track cells entering the tumor from the circulation, tumors were established in CD45.2 mice and blood from congenic CD45.1 mice was collected in heparin coated Eppendorf tubes and 100 µl of unmodified blood was adoptively transferred to tumor-bearing mice by retro-orbital injection. Tumors were harvested to identify bead^+^ or CD45.1^+^ cell infiltration and activation in the tumor by flow cytometry.

For combination therapy studies, mice were randomized to receive anti-CD80 (BioXCell clone 16-10A1) and anti-CD86 (BioXCell GL1) antibodies intraperitoneally (IP) at 200ug/200ul, on M/W/F for 6 doses, starting one day before radiation. Tumors were measured three times per week using digital calipers, and average diameter was calculated as (length x width)/2.

For analysis of tumor infiltrates, on day 3 or 10 tumors were weighed and minced into small fragments and then transferred into gentleMACS C tubes (Miltenyi Biotec, #130–093-237) containing enzyme digest mix (250U/mL collagenase IV, 30 U/mL DNAse I, 5 mM CaCl_2_, 5% FBS and HBSS). Minced tissue pieces were further dissociated using the GentleMACS tissue dissociator from Miltenyi Biotec, then quenched and washed into single cell suspensions as previously described [13]. Cells were counted using the Guava instrument (EMD Millipore) and analyzed by flow cytometry.

### Flow cytometry

The antibody panels for specific experiments are listed in Tables 1–2. Flow cytometry was performed as previously described [13, 14]. Briefly, cells were stained with Live/Dead Fixable stain in PBS, then washed and incubated with α-CD16/CD32 antibodies in FACS Buffer [1 × PBS + 2 mM EDTA + 2% FBS]. The cells were washed and stained with surface antibody cocktail prepared in FACS buffer and Brilliant Stain Buffer Plus (BD Horizon, #566,385). After surface staining, the cells were washed and fixed with BD Cytofix/Cytoperm solution (BD Biosciences, #554,722). Intracellular staining was performed using the FOXP3/Transcription Factor Staining Buffer Set (eBioscience, #0–5523-00) according to the manufacturer’s protocol. Finally, the cells were washed and resuspended in FACS buffer and analyzed using a BD Fortessa. Data obtained from flow cytometry assays were analyzed using FlowJo software (Tree Star Inc.) and OMIQ software.

**Table 1:** Antibody staining panel for mixed spheroid experiments

	Fluorophore	Marker
1	APC-Cy7	Live/Dead
2	AF700	MHC Class II
3	BV510	CD86
4	BV650	CD11b
5	BV421	Hoechst
6	PerCP/Cy 5.5	F4/80
7	PE/Dazzle CF594	CD80
8	PE-Cy 7	CD206
9	APC	CD69
10	PE	CD11c
11	FITC	Kaede

**Table 2:** Antibody staining panel for in vivo tumor analysis

		Tumor macrophages	Tumor microbeads	Congenic cell tracking	Treg analysis
	Fluorophore	Marker	Marker	Marker	Marker
1	BUV395		CD4	CD4	CD4
2	BUV496		CD103	CD103	CD103
3	BUV563		MHCII	MHCII	MHCII
4	BUV737		TCRB	TCRB	CD25
5	BV421	MHCII	CD80	CD80	CD80
6	Pacific Blue		Ki67		
7	BV510	Live/Dead	Latex Beads	CD45.1	
8	BV570		CD8		CD8
9	Zombie Yellow		Live/Dead	Live/Dead	
10	Zombie Aqua				Live/Dead
11	BV650	CD86	F4/80	F4/80	F4/80
12	BV711	Ly6C	CD44	CCR8	
13	BV785	CD45.2	CD45.2	CD45.2	CD45.2
14	FITC		CD11b	CD11b	CD11b
15	PerCP-eFluor 710		CD3	CD3	
16	PE	CD11c	ZAP70	FoxP3	Foxp3
17	PE-Dazzle594		CD11c	Ly6G	Ly6G
18	PE-Cy7	CD206	iNOS	iNOS	CD69
19	APC	CD103	Arg1	Arg1	CD86
20	AF700	CD90.2 + CD19	Ly6C	Ly6C	CD90.2
21	APC-eFuor 780		CD24	CD24	
22	PerCP-Cy5.5	F4/80		CCR4	
23	BUV661				Ly6C
24	eFluor 450				Ki67
25	BV605	CD11b			CD62L
26	APC-Cy7				CD44
27	PE-CF594	CD80			
28	APC-e780	CD24			

### Gene expression analysis

The relative expression of key genes in cells infiltrating MC38 tumors [15] and a panel of murine tumors [16] was analyzed using published public scRNASeq datasets. In patients, similar analyses used a published dataset of 371,223 cells from over 60 patients with colorectal cancer [17]. All scRNASeq data were analyzed using BbrowserX and Vinci software (BioTuring Inc., San Diego, CA, USA: https://academic.bioturing.com).

### Statistical analysis

Data were graphed and analyzed using Prism (GraphPad). Survival data were assessed using a logrank test. Individual parameter datasets across multiple groups were compared using a one-way ANOVA with multiple comparisons corrected using Tukey’s method. Individual parameter datasets across two groups were compared using an unpaired t test. Error bars represent SEM.

## Results

### Spheroid co-culture model

**Fig. 1:**
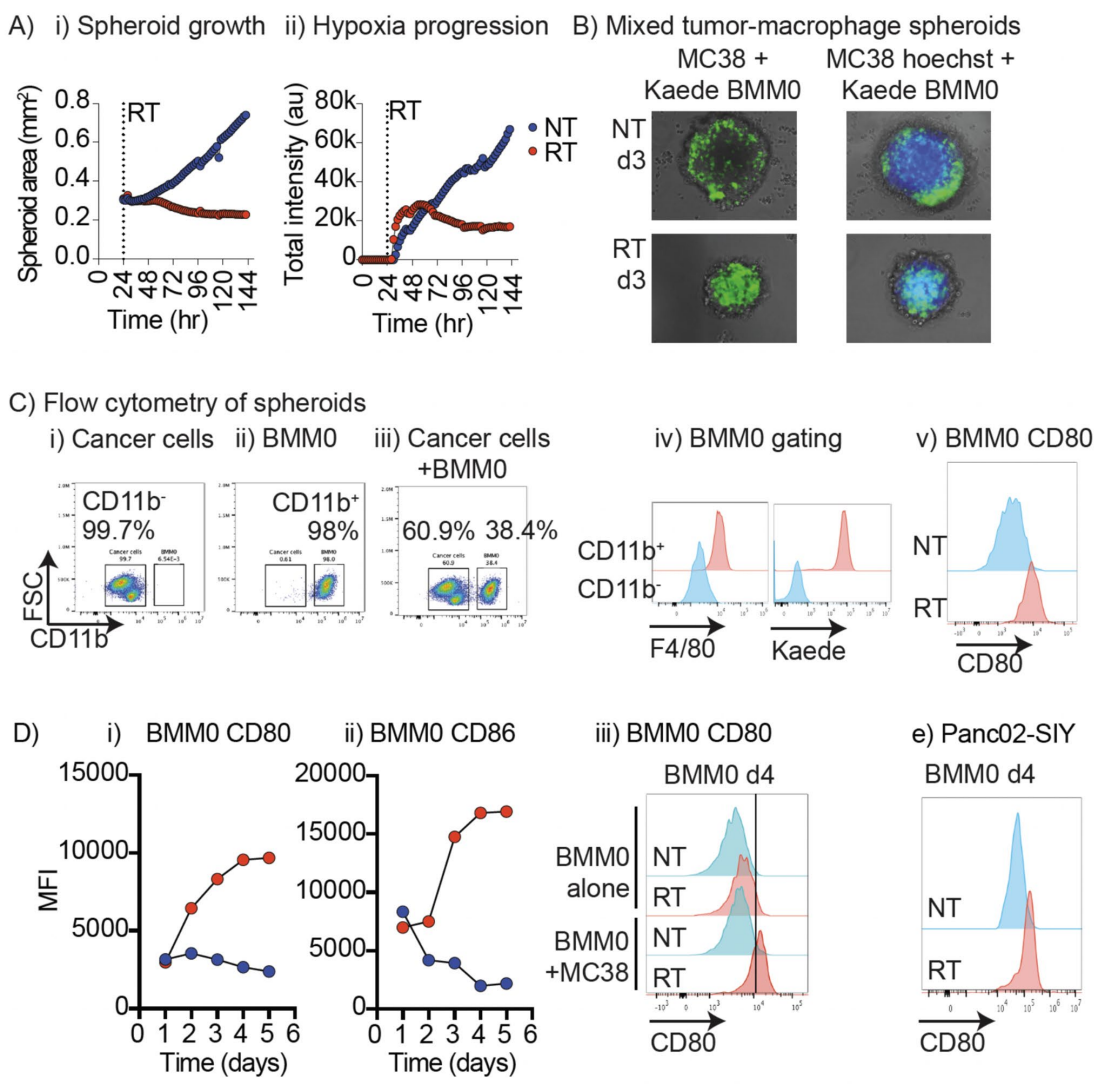
Macrophages in tumor spheroids are activated following radiation therapy. **A** MC38 tumor spheroids were established in vitro and at 24 h left untreated (blue) or exposed to 12 Gy of radiation (red). i) Spheroid cultures were followed using live cell imaging to assess spheroid size over a 6-day period. ii) Spheroids were treated with Image-iT Hypoxia reagent, and level of hypoxia was measured by fluorescence intensity using live cell imaging. **B** MC38 cancer cells left untreated or stained with Hoechst live cell dye (blue fluorescence) were co-cultured with bone marrow-derived macrophages from Kaede transgenic mice (green fluorescence) for 24 h. The resulting MC38-macrophage spheroid co-cultures were either left untreated or exposed to 12 Gy of radiation and the cultures were harvested on day 3 post radiation. Images show representative live cell images of untreated or irradiated mixed spheroids. **C** Spheroids were disrupted for flow cytometry, confirming the gating of i) CD11b- cancer cells seeded alone, ii) CD11b + macrophages seeded alone, or iii) cancer cells and macrophages seeded in combination. iv) Correct gating was confirmed by expression of the macrophage marker F4/80 and the Kaede transgene. v) Expression of CD80 in bone marrow macrophages from mixed tumor spheroids left untreated or 3d following treatment with 12 Gy RT. **D** Quantification of i) CD80 and ii) CD86 expression on bone marrow macrophages from mixed tumor spheroids left untreated or treated with 12 Gy RT over time. iii) Comparison of CD80 on bone marrow macrophages alone, or from mixed tumor spheroids left untreated or treated with 12 Gy RT. E. Mixed tumor spheroids were generated using the Panc02-SIY cancer cell line and bone marrow macrophages, left untreated or treated with 12 Gy RT, and analyzed for CD80 on macrophages on d4 post-RT. Results are representative of more than 3 independent experiments

To model for cancer-myeloid interactions without recruitment of new cells, we developed an ex vivo 3D culture model. MC38 cancer cells grown in an ultra-low attachment plate formed a spheroid. Untreated spheroids progressively grew (Fig. 1Ai) and 12 Gy radiation significantly impaired spheroid growth across a range of seeding cell numbers (Fig. 1Ai, Supplemental Fig. 1). To assess spheroid integrity, we used the Image iT reagent that fluoresces in low-oxygen environment. Hypoxia progressively increased in untreated spheroids, whereas irradiated spheroids lost their hypoxic core or failed to develop hypoxia, depending on the size at treatment (Fig. 1Aii, Supplemental Fig. 1). To incorporate immune cells, we generated BMM0 and added these with the cancer cells to form a mixed spheroid. To distinguish the macrophages and the cancer cells, we used BMM0 from Kaede transgenic mice that have a baseline green fluorescence signal [18] and pre-labeled the cancer cells with the fluorescent marker Hoechst. Following spheroid formation, we observe that macrophages form an outer layer of the spheroid, with limited penetration into the structure (Fig. 1B). This is reminiscent of tumors in vivo, where stromal macrophages can form a barrier adjacent to cancer cell nests, which in turn can have hypoxic cores.

To address the impact of radiation, we irradiated mixed spheroids and investigated cell distribution. Following radiation of the mixed spheroid, we observed macrophages penetrating the spheroid, concomitant with the destabilization of the spheroid structure (Fig. 1B). To assess the impact of radiation on macrophage phenotypes, we performed flow cytometry and the macrophages were distinguished based on expression of CD11b, F4/80, and the Kaede marker (Fig. 1 Ci-iv). We identified a significant upregulation of CD80 expression on BMM0 in the spheroid following radiation therapy (Fig. 1Cv). Time-course analysis demonstrated upregulation of CD80 and CD86 starting d2 following radiation (Fig. 1Di-ii). Upregulation of CD80 did not occur when macrophages were irradiated alone (Fig. 1Diii). Most cancer cells in untreated spheroids were viable, but following radiation, cancer cells lost viability (Supplemental Fig. 2A-B). Macrophages also showed a decrease in viability following radiation but were less sensitive to radiation than the cancer cells. Importantly, the activation of macrophages following radiation was not a model-specific phenomenon as spheroids of the pancreatic adenocarcinoma cell line Panc02-SIY combined with BMM0 also resulted in CD80 upregulation following radiation (Fig. 1E).

**Fig. 2:**
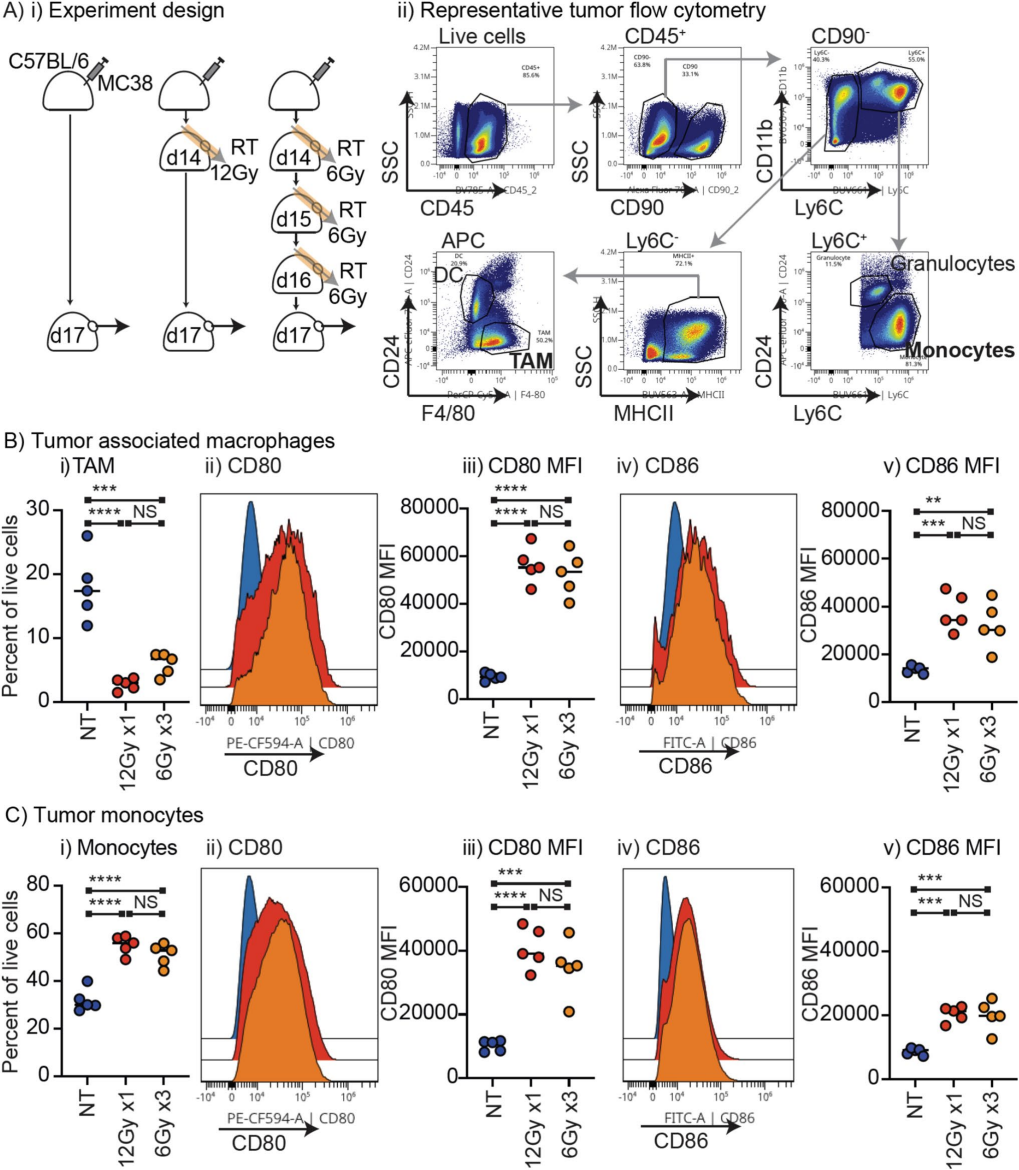
In vivo tumor macrophage and monocyte response to radiation therapy. **A** i) MC38 tumors were established in C57BL/6 mice and were left untreated or treated with 12 Gy radiation using CT guidance on day 14 post injection, or 3 daily doses of 6 Gy (d14,15,16). Tumors were harvested 3d (d17) following the first dose of radiation and single cell suspensions were analyzed by flow cytometry. ii) Preliminary gating strategy to characterize tumor-associated macrophages (TAM) as CD90-Ly6C-MHCII + CD24-F4/80 + cells, monocytes as CD90-Ly6C + CD24- cells. **B** i) TAM as a percent of live cells in the tumor, ii) representative histograms of CD80 in TAM in untreated or irradiated tumors. iii) quantification of TAM CD80 MFI in untreated or irradiated tumors. iv) representative histograms of CD86 in TAM in untreated or irradiated tumors. v) quantification of TAM CD86 MFI in untreated or irradiated tumors. **C** i) monocytes as a percent of live cells in the tumor, ii) representative histograms of CD80 in monocytes in untreated or irradiated tumors. iii) quantification of monocyte CD80 MFI in untreated or irradiated tumors. iv) representative histograms of CD86 in monocytes in untreated or irradiated tumors. v) quantification of monocyte CD86 MFI in untreated or irradiated tumors. (**p < 0.01; ***p < 0.001; ****p < 0.0001; NS = not significant). Results are representative of more than 3 independent experiments

To determine whether the in vitro response in 3D culture recapitulates the in vivo macrophage responses to radiation, we established MC38 tumors in C57BL/6 mice and treated the tumor with 12 Gy RT using CT guidance. To study the effect of different radiation delivery approaches, we also treated a group of tumors with 6 Gy × 3 focal radiation, which has a similar BED to 12 Gy × 1 (28.8 Gy vs 26.4 Gy, respectively). The tumors were harvested 4 days later (Fig. 2Ai) and analyzed by flow cytometry. Tumor-associated macrophages (TAM) were identified as CD45^+^CD90^−^Ly6C^−^CD24^−^F4/80^+^ cells using criteria defined by Broz et al*.* [19] (Fig. 2Aii). Notably, radiation resulted in CD80 and CD86 upregulation on TAM in vivo (Fig. 2B), consistent with the spheroid response. As observed in vitro, following RT we observed a decrease in the proportion of TAM in the tumor at this timepoint (Fig. 2B). This loss of TAM is outweighed by a large influx of CD45^+^CD90^−^Ly6C^+^CD24^−^ monocytes that occurs post-RT in vivo (Fig. 2C), and these cells also upregulate CD80 and CD86 following RT. There was no significant difference in the TAM or monocyte response between the two different RT approaches. These data validate that the ex vivo response of cancer cells and macrophages in 3D culture closely resemble the response seen in vitro.

### Role of innate activation pathways on macrophage responses to radiation

**Fig. 3:**
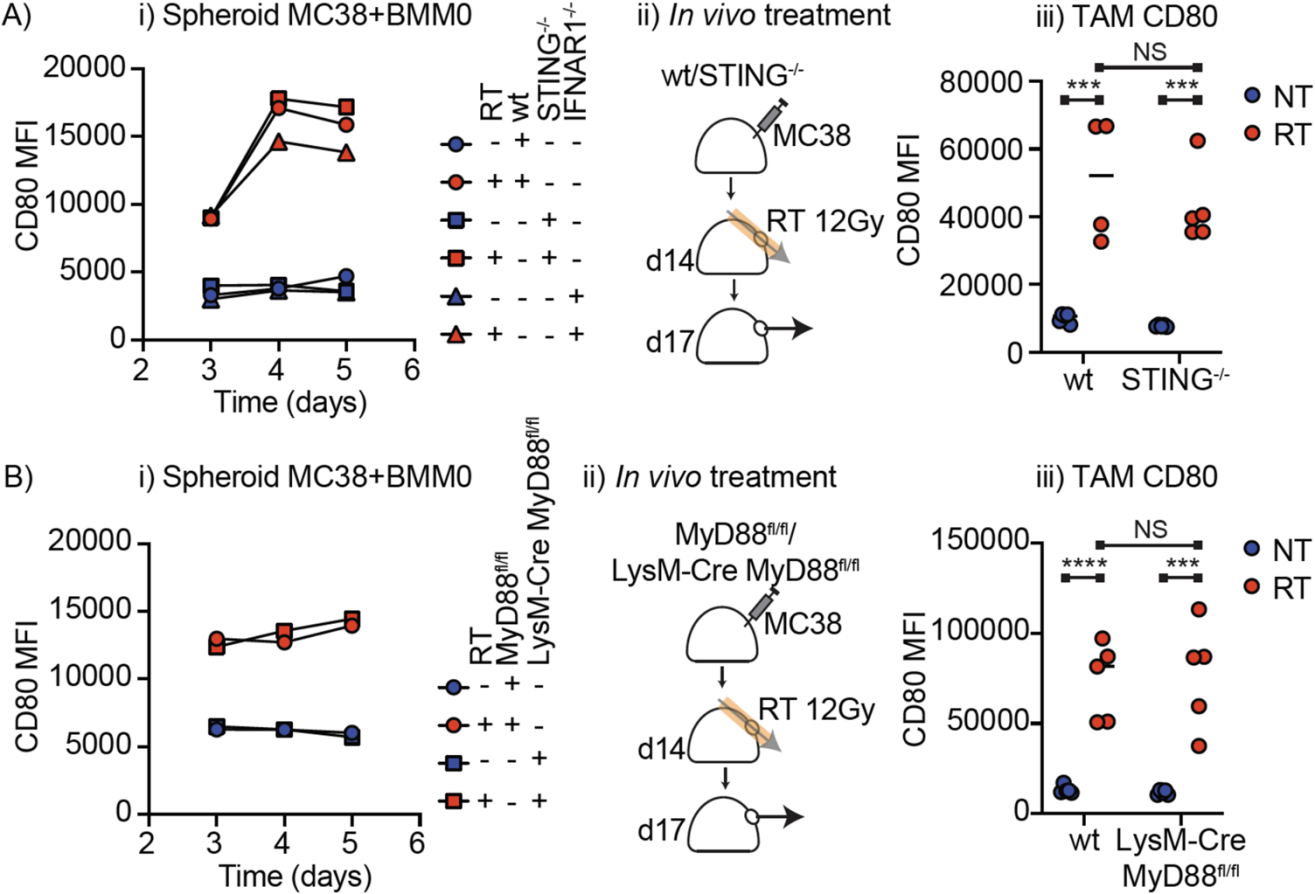
STING- and MyD88-independent macrophage activation i*n vitro* and in vivo. **A** Spheroids of MC38 cancer cells with bone marrow-derived macrophages from wild-type C57BL/6 mice, STING-/- mice or IFNAR1-/- mice were established for 24 h then left untreated or treated with 12 Gy RT. Spheroids were disrupted for flow cytometry and graphs show the expression of CD80 on the macrophages. ii) MC38 tumors were established in wild-type C57BL/6 mice or STING-/- mice and were left untreated or treated with 12 Gy radiation using CT guidance on day 14 post injection. The tumors were harvested 3d following radiation, and single cell suspensions were analyzed by flow cytometry. iii) Quantification of CD80 MFI on macrophages in untreated or irradiated tumors. **B** Spheroids of MC38 cancer cells with bone marrow-derived macrophages from MyD88fl/fl or LysM-Cre MyD88fl/fl mice were established for 24 h then left untreated or treated with 12 Gy RT. Spheroids were disrupted for flow cytometry, and graphs show the expression of CD80 on the macrophages. ii) MC38 tumors were established in MyD88fl/fl or LysM-Cre MyD88fl/fl mice and were left untreated or treated with 12 Gy radiation using CT guidance on day 14 post injection. The tumors were harvested 3d following radiation, and single cell suspensions were analyzed by flow cytometry. iii) Quantification of CD80 MFI on macrophages in untreated or irradiated tumors. (***p < 0.001; ****p < 0.0001). Results are representative of 2 independent experiments

Radiation has been shown to cause the release of STING ligands from irradiated cancer cells. This can impact macrophages and dendritic cells in the tumor in vivo and is a plausible mechanism for macrophage activation in the spheroid model. To address this, we generated spheroids of MC38 cancer cells combined with wild-type (wt) or STING^−/−^ BMM0. We observed a matched increase in CD80 expression in both wild-type and STING^−/−^ BMM0 (Fig. 3Ai), suggesting that activation of the STING pathway in the TAM is not necessary for this effect. It is possible that the MC38 cells are themselves sensing extranuclear DNA via cGAS and activating the STING pathway. This results in type I IFN release, which could be the mechanism for macrophage activation. To address this, the experiment was repeated with BMM0 that have a knockout in the type I IFN receptor (IFNAR1^−/−^). We similarly observed an increase in CD80 expression in IFNAR1^−/−^ BMM0 (Fig. 3Ai), demonstrating that TAM activation is not dependent on type I IFN. Consistent with data above, radiation of macrophages alone did not result in upregulation of CD80 (Supplemental Fig. 3). To validate these data in vivo, we established tumors in wt or STING^−/−^ mice, and the tumors were left untreated or treated with 12 Gy RT (Fig. 3Aii). Radiation resulted in CD80 upregulation on TAM (CD45^+^CD3^−^Ly6C^−^MHCII^+^F4/80^+^) in both wt and STING^−/−^ mice (Fig. 3Aiii), confirming that as in the spheroid model, upregulation of CD80 is independent of STING signaling in the macrophages.

The major alternative innate adjuvant signal released following radiation includes Toll-like receptor ligands that signal via MyD88. To address this, we established spheroids with BMM0 from MyD88^fl/fl^ or LysM-Cre MyD88^fl/fl^ mice. There was no significant impact of MyD88 loss on RT-induced macrophage activation (Fig. 3Bi), demonstrating that this is not the mechanism of macrophage activation by RT in spheroids. To validate these data in vivo*,* we established MC38 tumors in wt mice or LysM-Cre MyD88^fl/fl^ mice. Tumors were left untreated or treated with 12 Gy RT and the tumors harvested 3 days later (Fig. 3Bii). RT resulted in an upregulation of CD80 in TAM in both wt and LysM-Cre MyD88^fl/fl^ mice (Fig. 3Biii). These data demonstrate that consistent with the spheroid model, tumor-associated macrophages upregulate CD80 in a MyD88-independent manner.

### Role of direct radiation in CD80 upregulation.

**Fig. 4:**
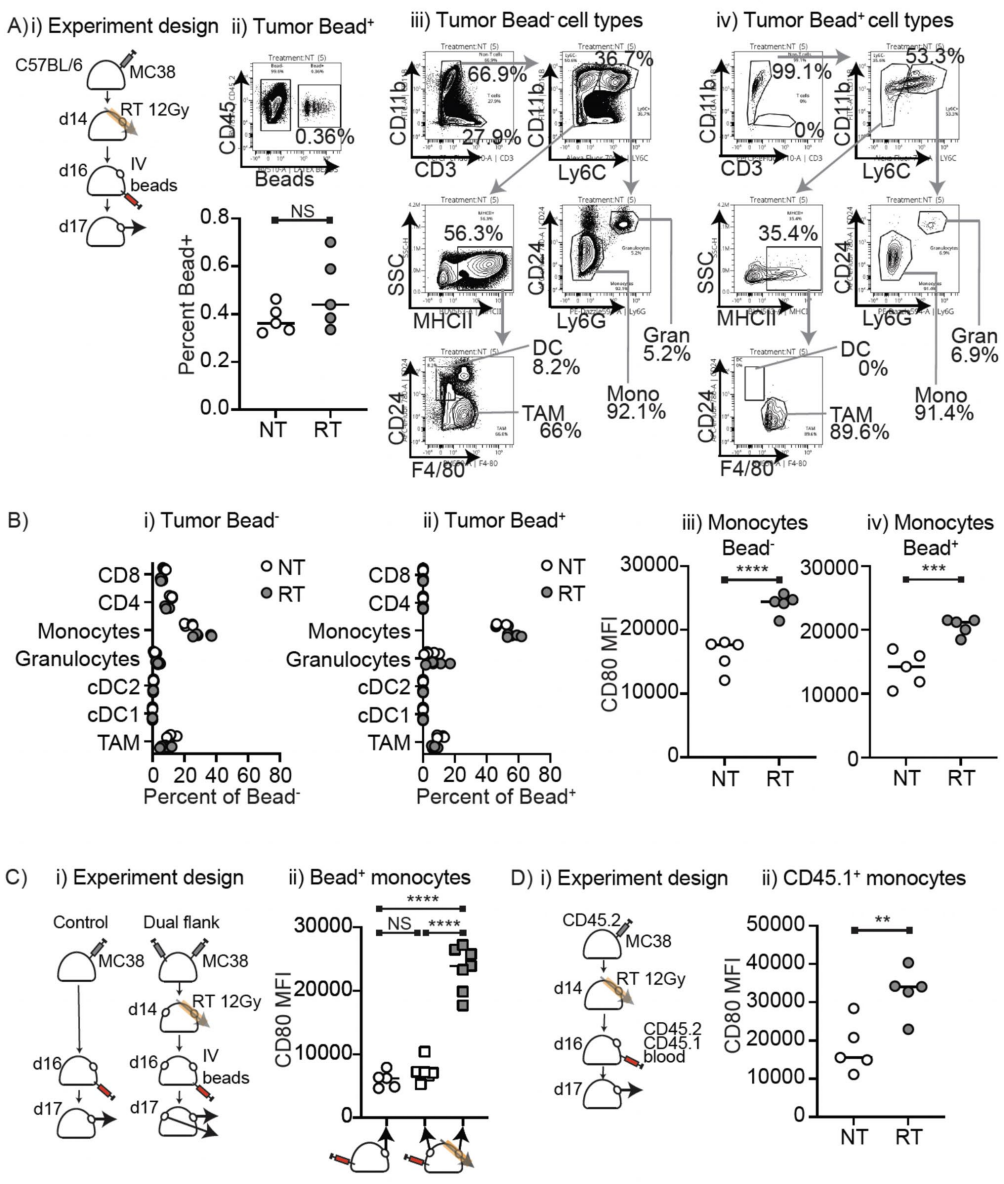
Cell tracking demonstrates the impact of the tumor environment on myeloid activation. **A** i) MC38 tumors were established in C57BL/6 mice were left untreated or treated with 12 Gy radiation using CT guidance on day 14 post injection. 2d post-RT, the mice were injected IV with fluorescent microbeads. Tumors were harvested 1d later, and single cell suspensions were analyzed by flow cytometry. ii) representative example showing identification of cells containing microbeads in the tumor and the percent bead + cells in NT versus RT tumors. Representative flow cytometry to identify the cell types that are iii) bead-ve versus iv) bead + ve. **B** Quantification of cell populations defined in A) in i) that are Bead-ve versus ii) bead + ve with NT (open circles) or RT (closed circles). iii) Quantification of CD80 MFI on monocytes that are iii) Bead-ve versus iv) bead + ve with NT (open circles) or RT (closed circles). **C** i) single or dual flank MC38 tumors were established in C57BL/6 mice, and the single flank tumor was left untreated and one of the dual flank tumors was treated with 12 Gy radiation using CT guidance on day 14 post injection. 2d post-RT, the mice were injected IV with fluorescent microbeads. Tumors were harvested 1d later, and single cell suspensions were analyzed by flow cytometry. ii) Quantification of CD80 MFI on Bead + monocytes. **D** i) MC38 tumors injected into CD45.2 congenic mice were left untreated or treated with 12 Gy radiation using CT guidance on day 14 post injection. 2d post-RT, the mice were injected IV blood from CD45.1 congenic mice. Tumors were harvested 1d later, and single cell suspensions were analyzed by flow cytometry. ii) Quantification of CD80 MFI on CD45.1 + monocytes. Key: NS = not significant; **p < 0.01; ***p < 0.001; ****p < 0.0001. Results are representative of 2 independent experiments

In the spheroid model and the in vivo model, macrophages are in the radiation field and so receive equivalent radiation to the cancer cells. However, macrophages are rapidly replenished in the tumor and at later time points there are increased macrophage numbers that can negatively regulate anti-tumor immunity and guide cancer recurrence. In vitro we see no impact of direct radiation on macrophage activation when cultured in the absence of cancer cells, but it is more difficult to validate whether the incidental radiation the macrophages receive is involved in their activation or is irrelevant. To exclude direct radiation, we developed a novel model to distinguish myeloid cells that have entered the tumor after radiation delivery. Fluorescent microbeads administered IV are too large to directly enter tissues but are taken up by circulating monocytes which can enter tissues [20]. Thus, by analyzing cells in the tumor that contain fluorescent microbeads we can distinguish recent entrants to the tumor environment. We injected fluorescent microbeads IV 2 days following radiation and analyzed the tumor 1d later (Fig. 4Ai). We observe a small but measurable influx of Bead^+^ cells into the tumor that is not significantly impacted by RT (Fig. 4Aii). We observed that the Bead^+^ cells are enriched for phagocytic myeloid cells, particularly CD11b^+^Ly6C^+^CD24^−^Ly6G^−^ monocytes in untreated and irradiated tumors (Fig. 4A-B). This is consistent with ongoing recruitment of circulating monocytic cells from the peripheral circulation to the tumor environment. Notably, the Bead^+^ monocytic cells recruited to irradiated tumors upregulate CD80 to a similar degree to the Bead^−^ cells (Fig. 4Biii-iv), demonstrating that recruited cells are induced to upregulate CD80 once in the tumor environment. It remains possible that circulating cells also received a radiation dose, so to address this we established dual tumors in mice and irradiated only one tumor. Mice were given IV treatment with beads on d2 post-RT, and we harvested both tumors on d3 post-RT (Fig. 4Ci). In this model, we see clear upregulation of CD80 on Bead^+^ monocytes in the irradiated tumor, but not on Bead^+^ monocytes in the non-irradiated tumor on the opposite flank (Fig. 4Cii), indicating that this is a site-specific and not a systemic effect of radiation.

Bead uptake tags only phagocytic cells in the circulation and risks altering phagocyte biology, though this has not previously been noted [20]. To confirm the impact of radiation on recruited cells using a different approach, tumor-bearing CD45.1^+^ mice were left untreated or treated with 12 Gy RT, then 2 days later received IV transfer of 50 µl of unmodified peripheral blood from CD45.1 congenic mice (Fig. 4Di). These transferred CD45.1^+^ congenic cells could be identified via their congenic marker and unlike the host CD45.2^+^ cells were not in the animal at the time of radiation. Analysis of the tumors 1d following transfer allowed us to identify a small population of transferred CD45.1^+^ cells in the tumor (Supplemental Fig. 4A), demonstrating that some of the transferred cells had migrated from the peripheral blood to the tumor environment. Again, the recruited population was dominated by monocytes (Supplemental Fig. 4B), and these cells upregulated CD80 in irradiated tumors but not in untreated tumors (Fig. 4Dii). Since these cells were in a different animal at the time of radiation, these data formally prove that the irradiated tumor environment rather than radiation results in CD80 expression in myeloid cells.

### Impact of CD80 and CD86 on overall survival following radiation therapy

CD80 and CD86 are potent costimulatory molecules that are critical for effective T cell activation and expansion. However, while macrophages and monocytes may be actively phagocytosing cancer cell materials, these cell lack the ability to cross-present antigens to CD8 T cells, a biology that is restricted to dendritic cells [21]. These data would imply that increased CD80 and CD86 on macrophage and monocytes would only be able to impact CD4 T cells via MHCII presentation. Of the CD4 T cells, Treg express high levels of CTLA4 [22, 23], therefore these cells are likely to be highly sensitive to CD80/CD86 ligation of CTLA4, which is a higher affinity ligand than CD28 [24, 25]. To better understand the susceptibility of T cells to costimulation through CD80 and CD86, we analyzed published scRNASeq data from T cells in MC38 tumors [15]. Both Ctla4 and Cd28 were variably expressed by different T cell populations (Supplemental Fig. 5), but as anticipated Ctla4 expression was enriched on T regulatory cells. CD28 was present in less activated Pcdc1^−^ (PD1^−^) cells as well as more exhausted Pcdc1^+^Havcr2^+^Lag3^+^ cells, while Ctla4 was not present in the less activated Pcdc1^−^ cells but was present in the more exhausted Pcdc1^+^Havcr2^+^Lag3^+^ cells (Supplemental Fig. 5). Thus, only the less activated CD8 T cells in the tumor have Cd28 expression that is unopposed by Ctla4.

To address alternative sources of CD80 and CD86 in the tumor environment, we profiled expression of these markers along with PDL1 (CD274) in published scRNASeq data from five different murine tumors including MC38 (Supplemental Fig. 6). We found that at baseline the cancer cells in vivo consistently lack CD80 and CD86, but can express PDL1, as anticipated. In these tumors macrophages are a consistent source of CD80 and CD86. To determine whether this was true in patients, we analyzed a published dataset of 371,223 cells from over 60 patients with colorectal cancer [17]. As in the murine models, cancer cells lack costimulation markers, and CD80 and CD86 expression is limited to myeloid populations in the tumors (Supplemental Fig. 6).

**Fig. 5:**
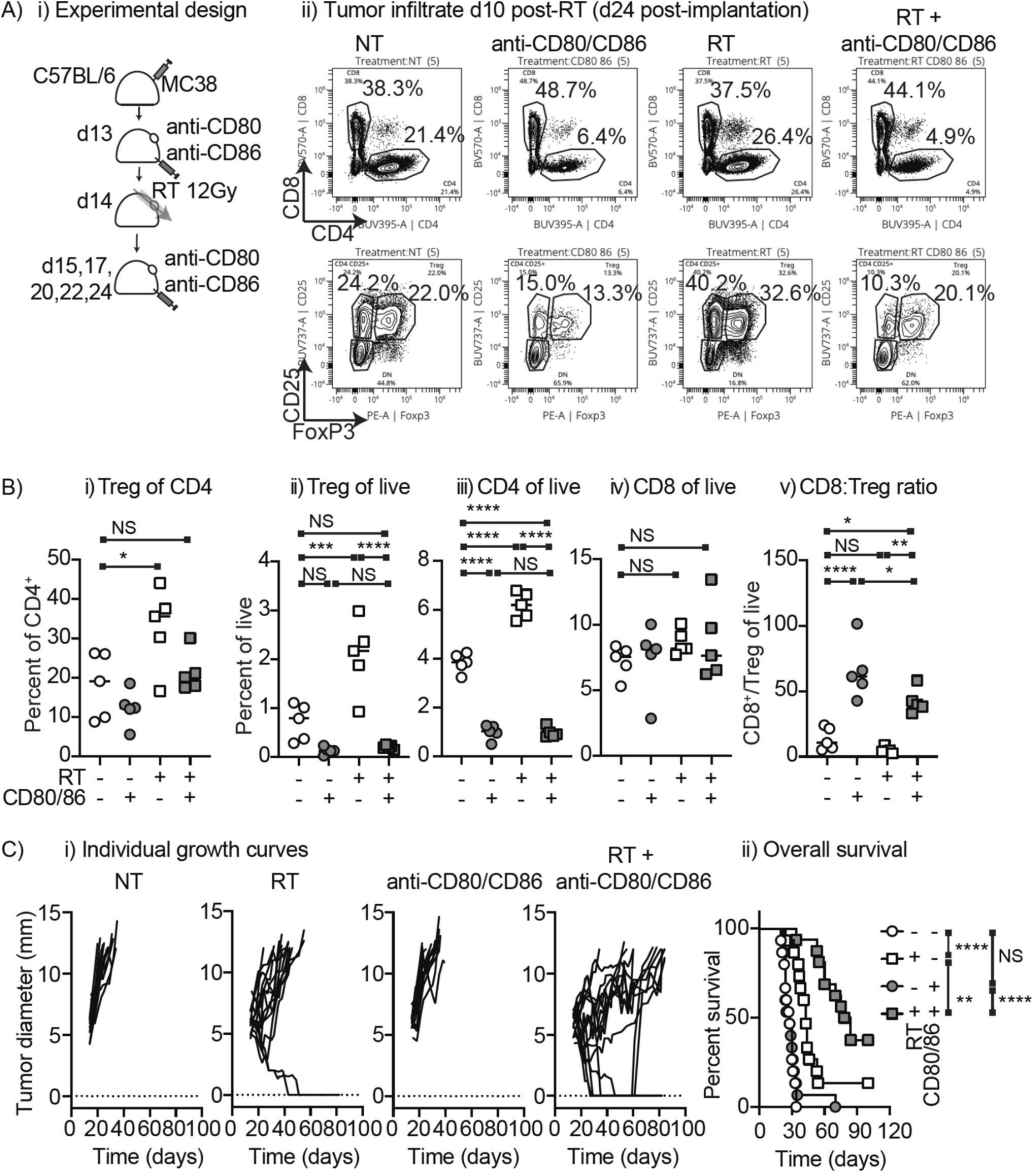
Costimulation blockade prevents Treg expansion post-RT and improves tumor control by RT. **A** i) MC38 tumors were established in C57BL/6 mice were left untreated or treated with 12 Gy radiation using CT guidance on day 14 post injection. Mice were also randomized to receive no further treatment or the combination of anti-CD80 and anti-CD86 blocking antibodies starting 1d prior to RT for six total doses over two weeks. ii) Tumors were harvested d10 post-RT and analyzed for immune infiltration. Graphs show representative gating for CD8 and CD4 T cells in live CD45 + CD90 + T cells, and FoxP3 + CD25 + Treg in gated CD4 T cells for each treatment group. **B** quantification of infiltrates identified in a) including i) Treg as a percent of CD4 and the percent of live cells in the tumor that are ii) Treg, iii) CD4, or iv) CD8 T cells. v) CD8:Treg ratio. **C** Mice were treated as in A. and followed for overall survival. i) individual growth curves where each line represents 1 tumor in 1 mouse. ii) overall survival. Key: NS = not significant; *p < 0.05; **p < 0.01; ***p < 0.001; ****p < 0.0001. Results are representative of 2 independent experiments

As Treg are the dominant CTLA4-expressing CD4 T cell that could productively interact with MHCII and CD80/86 expressing macrophages, it is notable that these cells have been shown to expand in proportion in the tumor at later timepoints following RT [26]. This timeline of Treg expansion by RT immediately follows our observed CD80/86 upregulation by RT, therefore we hypothesized that CD80 upregulation by RT suppresses anti-tumor immunity by causing Treg expansion. To test this hypothesis, we established MC38 tumors in C57BL/6 mice and randomized mice to treatment with RT, blocking Ab to CD80 and CD86, or the combination (Fig. 5Ai). We analyzed the tumors 10 days following radiation by flow cytometry. As anticipated, radiation therapy resulted in an increase in the proportion of CD4 T cells that were CD25^+^FoxP3^+^ Treg and an overall increase in Treg as a proportion of live cells in the tumor (Fig. 5A-B). Treatment with anti-CD80/CD86 blocked this radiation-mediated increase in Treg in the tumor. Notably, anti-CD80/CD86 as a single agent or in combination with radiation also resulted in an overall decrease in CD4 T cells as a percent of live cells in the tumor, without impacting CD8 T cell proportions. This results in a significant decrease in overall Treg numbers in the tumor, and a dramatic change in the CD8: Treg ratio (Fig. 5B). Thus, CD80 and CD86 are critical for CD4 T cells and for radiation-mediated Treg expansion in the tumor immune environment.

To determine whether this results in altered tumor control, the experimental setup was repeated, and mice were followed for outcome. Treatment with RT extended overall survival (Fig. 5C). While anti-CD80 and anti-CD86 treatment did not impact overall survival, the combination of RT with anti-CD80/CD86 treatment significantly extended survival compared to RT alone and compared to anti-CD80/CD86 alone (*p* < 0.01 and *p* < 0.0001, respectively) (Fig. 5C). We did not observe treatment-related toxicities in any treatment group. These data demonstrate that while radiation therapy upregulates costimulatory molecules in the tumor, costimulation limits tumor control following RT.

## Discussion

These data demonstrate using 3D ex vivo culture models and novel cell tracking approaches that the post-RT tumor environment locally activates myeloid cells. This upregulation of CD80 and CD86 on macrophages suppresses anti-tumor immunity following radiation therapy, despite the known importance of CD80 and CD86 in conventional costimulation. We demonstrate a mechanism of macrophage-driven immune suppression in the tumor environment post-RT via CD80 and CD86-mediated expansion of CD4 T cells and Treg.

Macrophages in the tumor environment play a multifaceted role following radiation therapy [27]. Macrophages are generally pre-polarized to immune suppressive phenotypes in the tumor [28, 29], such that exposure to innate adjuvants released on cell death drives anti-inflammatory rather than pro-inflammatory responses in the tumor environment [3, 12]. Thus, preventing molecular pathways associated with M2 polarization [28] improves tumor control by radiation [12]. Mertk is a phagocytic driver of M2 polarization [30], and loss of Mertk improves tumor control by radiation [2] via CD8 T cells [31]. Relatedly, macrophage phagocytosis of dying cells can result in TGFβ secretion [32], and TGFβ limits T cell control of tumors following radiation [33, 34]. Arginase is expressed by macrophages that polarized to suppressive phenotypes [35–37], and macrophage-selective loss of arginase improves T cell control of tumors following radiation [38]. Thus, while our data demonstrates upregulation of CD80 and CD86 on macrophages following radiation, this occurs as part of a much wider program of macrophage-driven immune suppression following radiation therapy. It is notable that CD80 upregulation occurs following treatment of unpolarized macrophages with LPS and IFNγ, while treatment with IL-4 does not result in CD80 upregulation [39]. Similarly, CD86 is upregulated following IFNγ and LPS polarization of macrophages but not following IL-4 polarization [40]. Given these studies, our data would suggest that radiation is driving a pro-inflammatory polarization of macrophages and monocytes in the tumor immune environment. It is possible that this early pro-inflammatory differentiation precedes the anti-inflammatory and pro-resolution differentiation of macrophages in irradiated tumors that is associated with later suppression of T cell responses [38, 41]. It would be valuable to further characterize the differentiation of these macrophages and the impact the interactions with Treg and CD4 via CD80 and CD86 have on the subsequent differentiation of the macrophages following radiation.

Since starting the work described in this manuscript, Frijlink et al. [42] demonstrated that blockade of CD80/86 improves tumor control by radiation therapy in the TC1 prostate tumor model, with the dominant action provided by CD86. This work also demonstrates that anti-CD86 blocks the Treg expansion in the tumor caused by radiation therapy [42], consistent with our data. In this work, the source of CD80 and CD86 upregulation is not determined. We would propose that myeloid cells likely link the mechanisms of radiation and CD80/86 mediated Treg expansion in these important studies. Together, these data strongly demonstrate a potential value to interventions blocking costimulation to improve tumor control following radiation therapy.

CD80 and CD86 blockade would also be expected to limit costimulation of other T cells through CD28, both locally and systemically. The fact that anti-CD80/86 has a benefit in combination with radiation suggests that at this stage of tumor therapy costimulation through CD28 is not as important as we would anticipate. This is supported by recent studies, which have shown that blockade of CD80 and CD86 following immunotherapy can improve outcomes in preclinical cancer models [43]. In addition, Zhou et al. demonstrated that blockade of CD80 and CD86 reduced the number of peripheral Treg in mice and these agents alone improved immune-mediated control of prostate and colorectal tumor models [44]. Mok et al. demonstrated that either combined anti-CD80/86 blocking antibodies or CTLA4-Ig following anti-CTLA4 immunotherapy improved tumor control [43]. This was associated with a reduction in Treg proportions in the tumor, and the authors similarly conclude that costimulation by CD80 and CD86 is more important for Treg than for the other T cells involved in tumor control. Relatedly, Liu et al. recently demonstrated that while CTLA4-Ig blocked the efficacy of anti-CTLA4 therapy, variants of CTLA4-Ig that did not bind anti-CTLA4 did not impair immunotherapy efficacy, despite binding and blocking CD80 and CD86 [45]. These CD80/86 selective variants also did not impair immunotherapy with anti-PD1 or anti-PD1 anti-CTLA4 combinations while they did reduce immune-related adverse events [45], indicating that any positive costimulation through CD80 and CD86 is unnecessary for the anticancer efficacy of these immunotherapies. Importantly, existing memory T cells are less dependent on costimulation for expansion in response to antigen than naïve T cells [46]. Using a conditional knockout model where CD28 is deleted following initial naïve T cell activation and expansion, Linterman et al*.* demonstrated that maintenance of virus-specific Th1 T cells is unchanged in the absence of continued CD28 expression, and clearance of virus is unimpacted by loss of CD28 [47]. However, expansion of all CD4 T cells and the maintenance of Tfh and Treg was shown to be dependent on continued CD28 expression [47]. Similarly, a range of antibody and genetic tools demonstrated that CD28 is important for secondary expansion of CD8 T cells, but not their development of effector function [48]. Thus, loss of costimulation through CD28 may not impact effector CD8 T cell control of residual cancer cells following radiation.

In the studies performed by Frijlink et al. [42] and in our studies reported here, CD80 or CD86 blockade had minimal therapeutic impact in the absence of radiation. Similarly, CD80 and CD86 blockade has been shown to improve tumor control along with, or following PD1 or CTLA4-targeted immunotherapy, but not alone [45]. The common factor in each of these examples is that the additional intervention, whether radiation or immunotherapy, has a positive impact on the tumor environment but can also cause a secondary negative effect mediated by Treg expansion. In these scenarios, blocking Treg expansion is more impactful than the anticipated negative effects of blocking costimulation through CD28. Based on our data and the data of Frijlink et al. [42], we propose that macrophages impact Treg via upregulation of CD80 and CD86, but at present this mechanism is not proven. This could be addressed in future experiments with targeted loss of CD80 and CD86 in macrophages, or though depletion of macrophages. However, current macrophage depletion strategies such as clodronate liposomes or CSF1R inhibitors result in only partial depletion [41, 49], and while they improve tumor control by radiation they are not curative therapies in preclinical models [41, 49]. By contrast Treg depletion alongside radiation therapy is highly effective in preclinical models [7, 50, 51]. As discussed above, macrophages have an array of potentially positive or negative impacts on the tumor [6, 27], but if macrophages are responsible for Treg expansion then this may be a major impact on the post-treatment immune environment of the tumor. Our studies have only been performed in female mice, and additional studies using male cancer cell lines in male mice would be justified to further validate the work.

Our in vivo and in vitro data demonstrate that CD80 upregulation on macrophages and monocytes is independent of their expression of MyD88, STING, and IFNAR1. Several papers suggest that cytokines may play a role in CD80 regulation. Both IFNγ and Type I IFN have been shown to upregulate CD80 [52, 53], though depending on the cell type and conditions, TNFα has been able [54] or unable [52, 53] to upregulate CD80. We and others have observed type I IFN expression increased in the tumor environment following radiation at the same timeline as CD80 induction [13]. However, increased type I IFN expression commonly depends on the same STING or MyD88-dependent adjuvant stimulation pathways that we show here are not needed for CD80 induction, and we show that IFNAR1 is unnecessary in macrophages to upregulate CD80. At present, we do not know the molecular mechanisms that drive CD80 and CD86 signaling in macrophages in the tumor. Though we do not know the specific mechanism, it is possible that these pathways can be redundant and for example upregulate cytokine production through STING if MyD88 is lost, and through MyD88 if STING is lost. Thus, radiation-induced CD80 upregulation on myeloid cells in the tumor environment may be present in a range of inflammatory scenarios dependent on the adjuvant and inflammatory triggers that are present, and each may support Treg expansion.

Together, our data demonstrate that radiation therapy causes upregulation of costimulatory molecules in myeloid cells, which supports Treg expansion following RT. These data link myeloid activation to Treg expansion following radiation and may guide interventions to regulate the complex dynamics limiting post-treatment immune control of residual cancer cells.

## Supplementary Information

Below is the link to the electronic supplementary material.

Supplementary file1 (DOCX 3196 KB)

## Data Availability

All data is present in the manuscript and supplemental figures.
