# Supplementary material for: Radiation therapy causes a STING and MyD88-independent upregulation of CD80 and CD86 in macrophages and monocytes that limits tumor control
Source: Cancer Immunol Immunother. 2025 Dec 19;75(1):21. doi: 10.1007/s00262-025-04272-0 (PMC12717329; doi:10.1007/s00262-025-04272-0)
Supplement: Supplementary file 1 — Supplementary file1 (DOCX 3196 KB) [file 262_2025_4272_MOESM1_ESM.docx]

**Supplementary Figures**


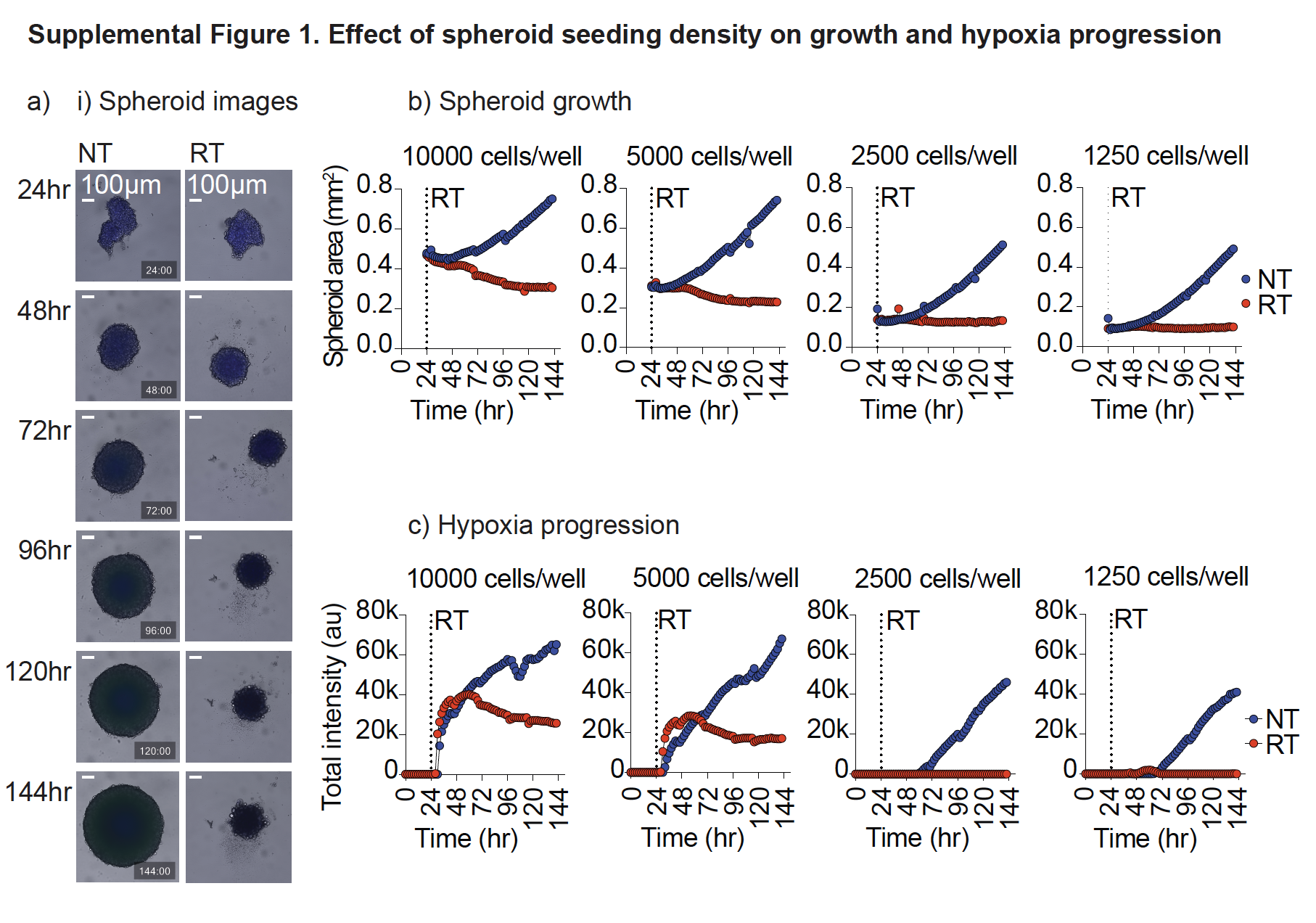


**Supplemental Figure 1.**

a) Representative images of MC38 tumor spheroids over time showing growth of untreated spheroids and growth arrest of spheroids treated with 12Gy. b) Spheroids were established in vitro at a range of seeding cell numbers and at 24 hours left untreated (blue) or exposed to 12Gy of radiation (red). Spheroid cultures were followed using live cell imaging to assess over a 6-day period. c) Spheroids were established as in b) and treated with Image-iT Hypoxia reagent and the level of hypoxia was measured by fluorescence intensity using live cell imaging.


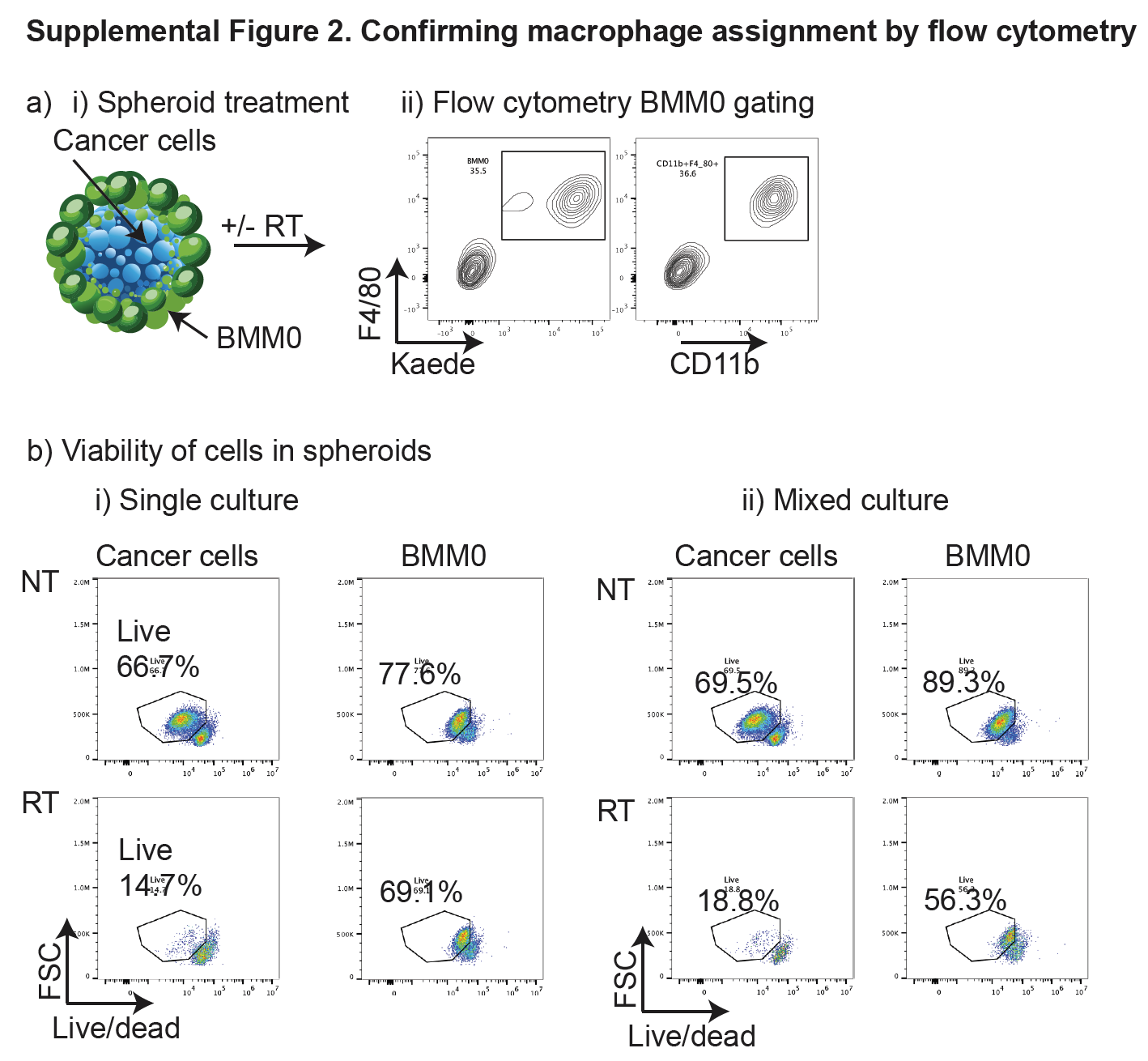


**Supplemental Figure 2.**

a) MC38 cancer cells left untreated or stained with Hoechst live cell dye (blue fluorescence), and were co-cultured with bone marrow-derived macrophages from Kaede transgenic mice (green fluorescence) for 24 hours. The resulting MC38-macrophage spheroid co-cultures were either left untreated or exposed to 12Gy of radiation and the cultures were harvested on day 3 post radiation. ii) Spheroids were disrupted for flow cytometry and cancer cells and macrophages were distinguished based on the expression of the macrophage markers CD11b, F4/80 and the Kaede transgene. b) Viability of the gated cells was assessed by dye exclusion in i) cancer cell spheroids or macrophages cultured alone, or ii) each cell type separately in mixed spheroid cultures.


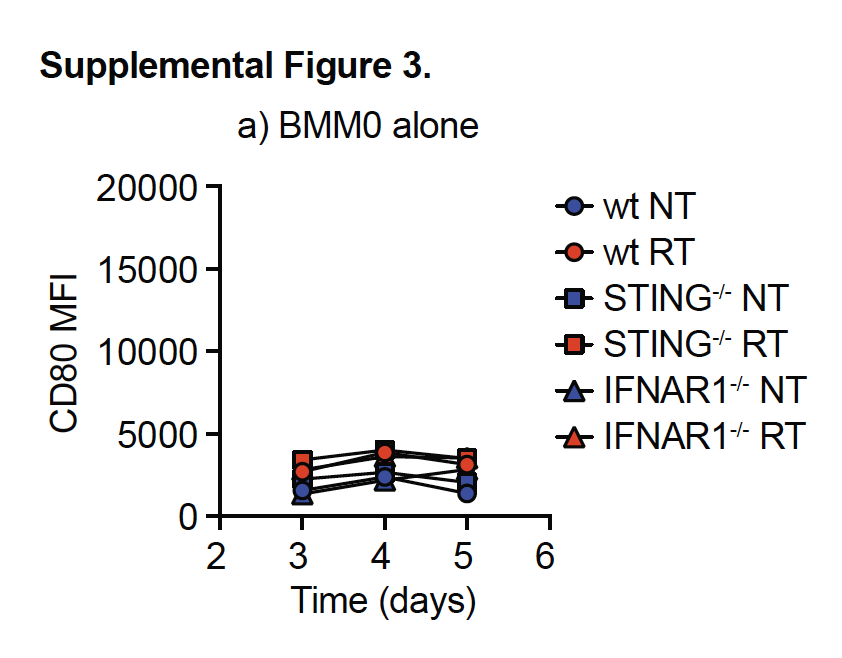


**Supplemental Figure 4.**

a) Bone marrow-derived macrophages from wild-type C57BL/6 mice, STING^-/-^ mice or IFNAR1^-/-^ mice were established for 24hr then left untreated or treated with 12Gy RT. Clusters were disrupted for flow cytometry and graphs show the expression of CD80 on the macrophages.


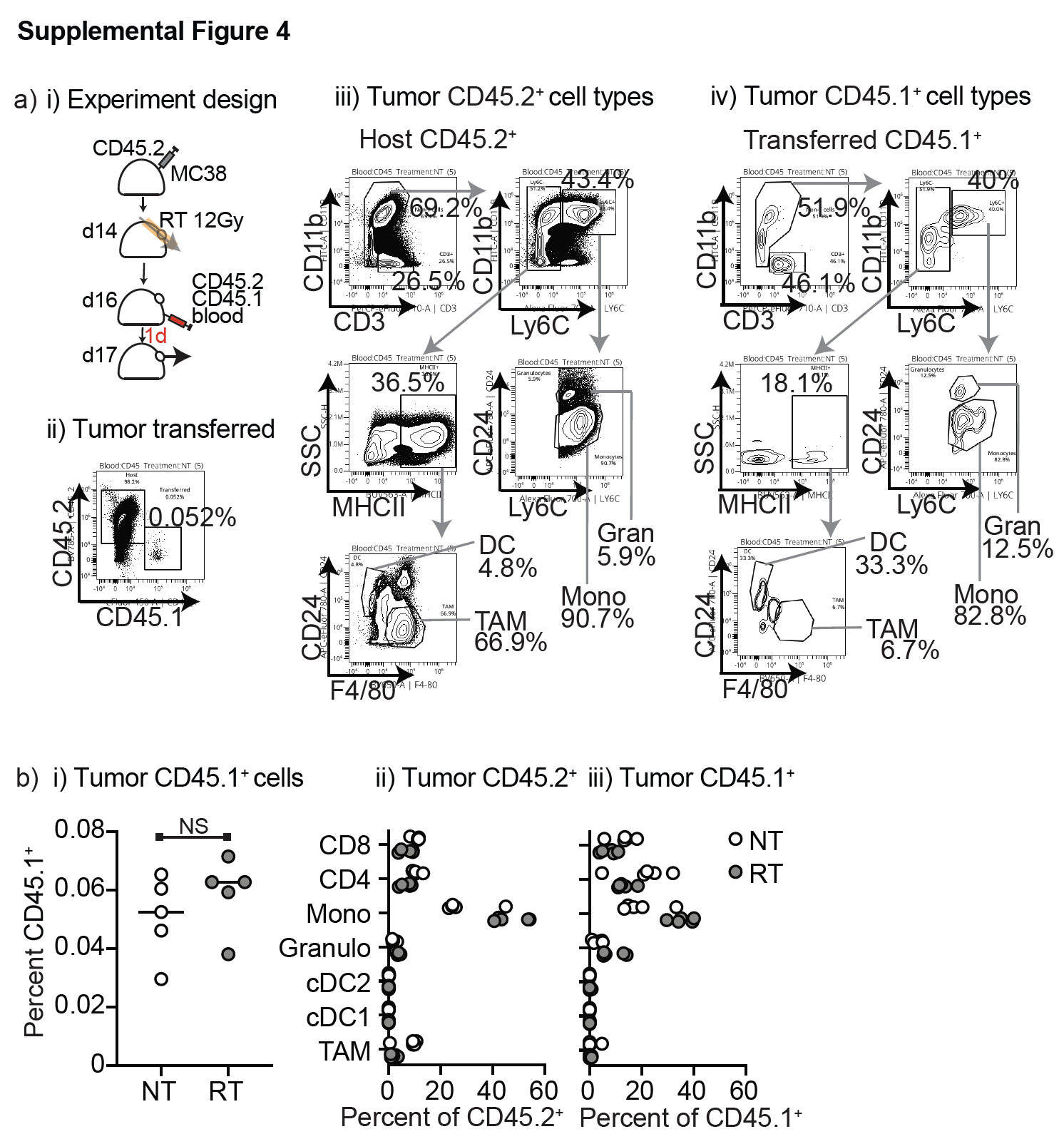


**Supplemental Figure 4.**

a) i) MC38 tumors injected into CD45.2 congenic mice were left untreated or treated with 12Gy radiation using CT guidance on day 14 post injection. 2d post-RT, the mice were injected IV blood from CD45.1 congenic mice. Tumors were harvested 1d later, and single cell suspensions were analyzed by flow cytometry. ii) representative example showing identification of CD45.1+ cells in the tumor. Representative flow cytometry to identify the cell types that are iii) CD45.2+ versus iv) CD45.1+. b) i) Quantification of CD45.1+ cell populations and the proportions of each major cell type that are ii) CD45.2+ versus ii) CD45.1+ in NT (open circles) or RT (closed circles) tumors. Key: NS = not significant


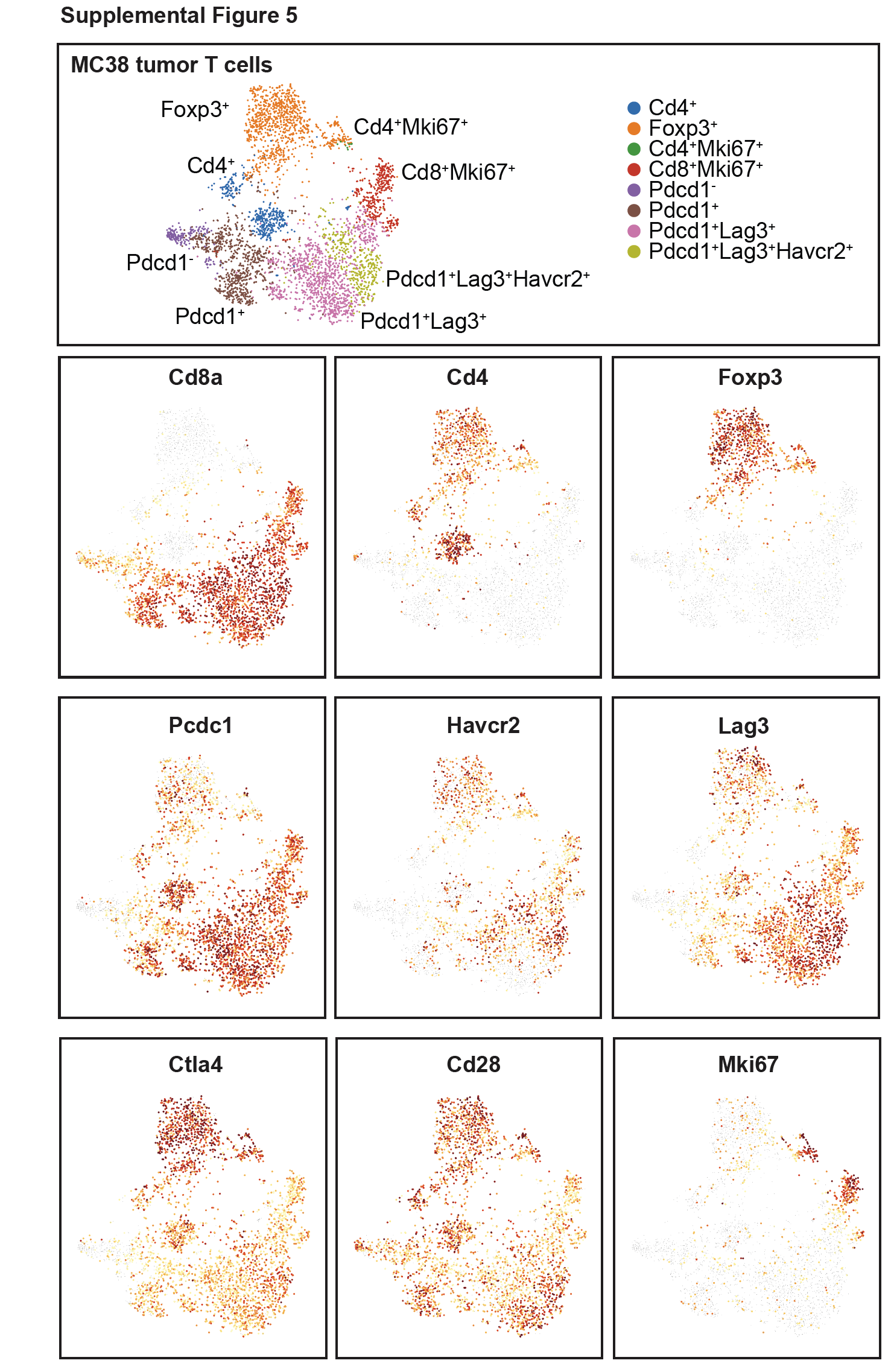


**Supplemental Figure 5. Expression of relevant molecules in T cells infiltrating MC38 tumors.**

Data from a public scRNASeq dataset of MC38 tumors was analyzed using BBrowserX (BioTuring Inc, San Diego, CA, USA) for the expression of the cell type defining markers Cd8a, CD4, and Foxp3, the exhaustion markers Pcdc1 (PD1) Havcr1 (Tim3), and Lag3, Ctla4, Cd28, and the proliferation marker Mki67. Graphs show author-defined cell subsets and the degree of each gene expression across all populations.


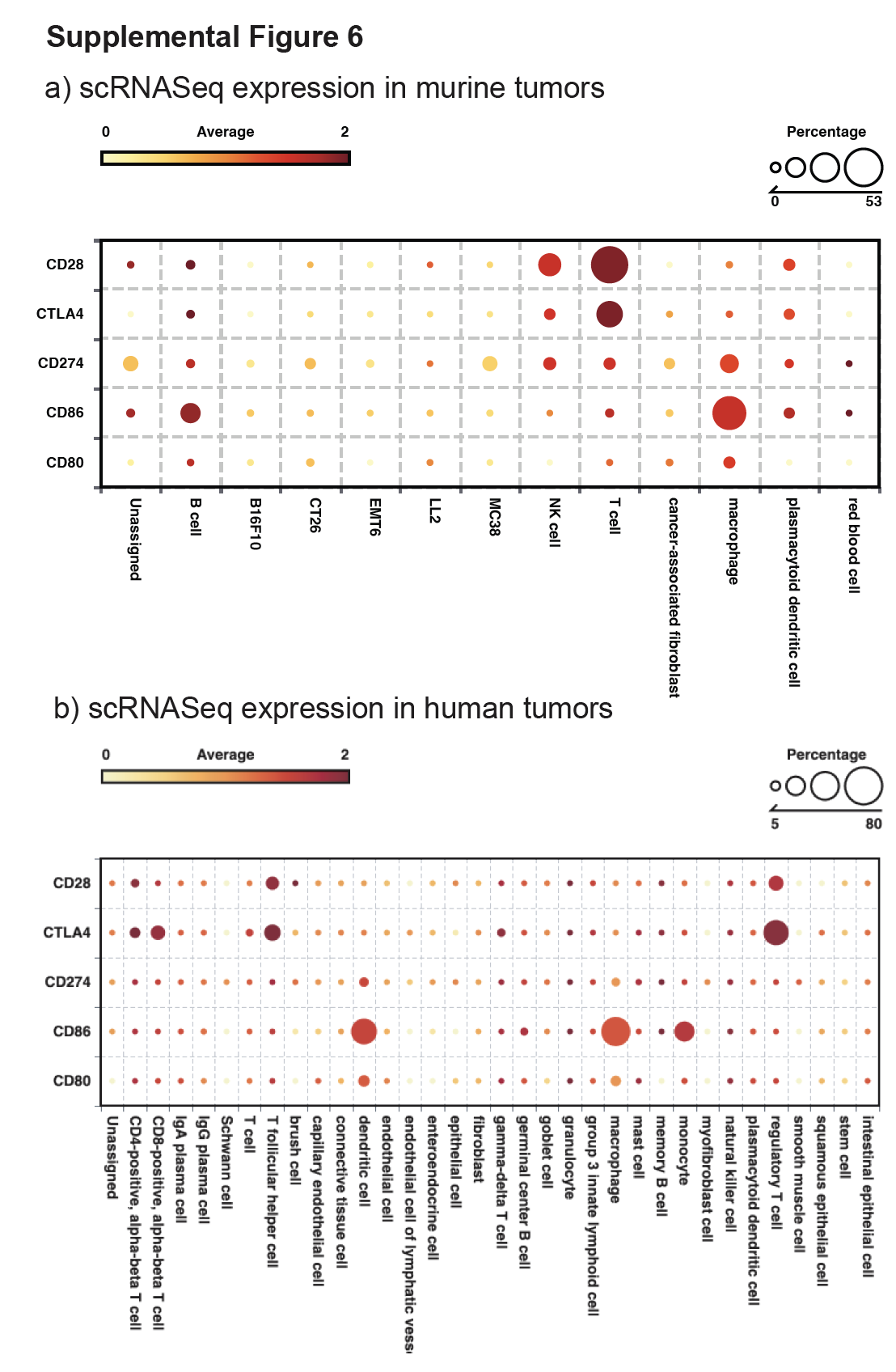


**Supplemental Figure 6. Expression of relevant molecules in murine and human tumors.**

Data from public scRNASeq datasets of a) murine and b) human tumors were analyzed using BBrowserX (BioTuring Inc, San Diego, CA, USA) for their expression of CD28, CTLA4, CD274 (PDL1), CD86, and CD80 in cell types defined by the originators. Circle size denotes the percentage of each cell type expressing the genes, and the color represents normalized average expression. Figures were created with Vinci software (BioTuring Inc, San Diego, CA, USA).
